# Construction of Evaluation Index System for Training Quality of High-Level Tennis Team

**DOI:** 10.3389/fpsyg.2022.902516

**Published:** 2022-06-29

**Authors:** Qian Cai, Yan Cheng, Yong Ke

**Affiliations:** ^1^School of Graduate, Wuhan Sports University, Wuhan, China; ^2^School of Tennis, Wuhan Sports University, Wuhan, China

**Keywords:** training quality, high-level tennis team, index system, real-time monitoring, comprehensive evaluation

## Abstract

The paper established the evaluation index system of training quality of high-level tennis team, which comprising five first-class indexes: work planning evaluation index (*i* = 4) includes realistic state diagnosis, training goal establishment, specific training content, and training plan formulation; work implementation evaluation index (*i* = 4) includes training guarantee conditions, training plan implementation, training process monitoring, and training plan adjustment; work monitoring evaluation index (*i* = 5) includes physical function, physical fitness, sports technique, sports tactics, and sport psychology; the evaluation index of team work management (*i* = 2) is the team training management and team organization management; and the competition performance index (*i* = 2) includes the results of large-scale events and the trend of sports talents. Each index of the system was obtained by sorting analysis of radar chart. The weight table of evaluation index was calculated by analytic hierarchy process. Therefore, the evaluation index system of training quality of high-level tennis team constructed in this study aims to provide reference for real-time monitoring, comprehensive evaluation, and diagnostic evaluation of each link of high-level tennis team training.

## Introduction

Training quality is an important factor to measure the improvement of athletes' competitive ability and affects not only their comprehensive performance, which is the core content for diagnosing and evaluating athletes, but also a comprehensive concept, which is composed of the coaches “coaching ability, training conditions, athletes” enthusiasm to participate in training and other elements. In the process of constructing and developing high-level sports teams, scientific and systematic training is the key link to change athletes' competitive ability and improve training performance, which plays a supporting and leading role in the cultivation and development of sports reserve talents. Since the scientific evaluation system can reflect the development of sports and the implementation effect of relevant policies, relevant workers can grasp the development process of training at any time. Many developed countries have studied this from the perspective of athletes, coaches' evaluation, and sports team management. The quality evaluation of sports team training includes three aspects: first, the perspective of athletes, including the physical condition, technical and tactical ability, pre-game state adjustment ability, and self-analysis of sports performance; second, the perspective of the coach, including the evaluation of teaching ability; and third, the angle of the sports team, mainly involving the team management (Lago-Ballesteros and Lago-Peoas, 2010; Neil et al., 2011; Surujlal, 2013; Ke, 2014; Zhuo et al., 2017; Huang, 2018; Zheng et al., 2019). However, the research on comprehensive evaluation of high-level tennis team is relatively weak, which fails to fully reflect the practical problems of training quality of high-level tennis players.

The “2011–2020 Olympic glory plan outline” issued by the State Sports Administration of China covers three aspects: strengthening the control of sports training process, drawing lessons from the modern quality management concept, and formulating and implementing the evaluation method of sports team training quality management. In view of the whole process of training and competition, qualitative and quantitative evaluation is carried out from the aspects of organization and management, planning and programming, training organization, goal realization, training innovation, competition command, combination of science and training, anti-doping, team culture, and echelon construction so as to improve the quality and efficiency of training (General Administration of Sport of China, 2015). Thus, consider the research and establishment of multi-disciplinary comprehensive training quality monitoring system as one of the key scientific research projects organized and supported by the end of the twenty-first century. Therefore, as the main driving force to promote the development of tennis competition in China, it is of great significance to ensure the training quality evaluation scientificity, rationality, and feasibility of the effective training of high-level tennis teams.

Finally, based on the perspective of training quality evaluation, using methods of expert interview, mathematical statistics, etc. to comb the current situation of China's high-level tennis team training from the micro level and clarify the influencing factors of the training quality of China's high-level tennis team from the macro level is implemented. On this basis, the evaluation index system of the training quality of the high-level tennis teams is constructed.

In the context of the hot development of Science–Technology–Engineering–Arts–Mathematics (STEAM) education, the all-round development of young people is of great concern. The construction of a high-level tennis team training quality evaluation system, from the dimension of scientific and technical literacy, has laid a good development space for the promotion of physical and mental health and the improvement of competitive ability of this special group of young athletes, and broadened the reserve of competitive reserve talents.

The purpose of this article is to provide a comprehensive evaluation method for the training quality of high-level tennis teams and to provide a theoretical basis for the scientific and reasonable regulation and control of the training work of high-level tennis teams.

## Literature Review

In the research of training quality evaluation, experts and scholars mainly focus on the process of mathematizing the evaluation contents of different sports with multiple objectives and criteria, and then obtain accurate and scientific evaluation results, which can be used as a guide and reference for improving athletic ability of athletes.

In the study of athletic ability of athletes, experts and scholars mainly focus on the characteristics of sports, highlighting the characteristics and complexity of competitive ability.

In the research on the evaluation index system of sports teams, Chinese scholars mostly discuss both subjective and objective aspects, and some studies present the analysis of a certain aspect, but it is not common to analyze the whole aspect of sports team training. Foreign scholars' research on the training of high-level sports teams mainly focuses on a particular aspect of the components of sports teams, such as sports techniques and tactics, athlete psychology, and other perspectives, and analyzes the problems related to the training of the whole sports team. As the rapid development of modern competitive sports has put forward higher requirements for sports team training, further research on systematic training of high-level sports teams is needed in order to make the training of sports teams develop in an efficient and refined way.

## Research Framework and Research Methodology

### Define the Scope and Construct the Framework

Through consulting and sorting out the literature on the quality of tennis team training, including journal papers, master's thesis, books, and relevant policy documents, the key words of “tennis team” and “training quality” were searched, summarized, and analyzed. Based on this, relevant experts' research of training quality of high-level tennis team are determined and divided into training work planning, training work implementation, training work monitoring, tennis team work management, and sports competition results.

### Field Research and Interview, Extract Indexes

Exploring the components, connotation, evaluation principles, methods of Training Quality of High Level Tennis Team, we have interview some experts through the way of face to face talk, telephone interview, and E-mail. The experts are mainly come from experts and scholars in sports colleges, front-line coaches work in provincial team and management personnel of all kinds of sports team. Based on the investigation of the current training situation of China's high-level tennis teams, the actual development of training planning, training implementation, training work monitoring, tennis team work management, and sports competition results are summarized and refined in Table 1.

**Table 1:** The basic information of the investigation experts.

**Category**	**Classification**	**Number**	**Percentage**
Education level	Doctoral Students	2	11.1%
	Master's Students	9	50%
	Undergraduate Students	7	38.9%
Positional titles	Professor	2	11.1%
	Associate Professor	9	50%
	Senior Instructor	7	38.9%
Areas of practice	Tennis Coaches	8	44.4%
	School Teachers	10	55.6%
	Scientific Researchers	0	0

According to the information in Table 1, it can be seen that the experts in the questionnaire are all with bachelor's degree or above, and their percentages are 38.9% for bachelor's degree, 50% for postgraduate degree, and 11.1% for doctoral degree, reflecting that the experts have higher education. Third, the experts in the questionnaire were all from tennis coaches and tennis teachers, with the percentages of 44.4 and 55.6%, respectively.

### Sort Out the Primary Indexes and Design the Questionnaire

According to the first two parts, sorting out the primary selection indexes of training quality evaluation of high-level tennis team and designing the questionnaire “screening table of training quality evaluation index of high-level tennis team.”

### Expert Authority Level

The degree of authority of the experts is an indication of the reliability of the questionnaire, which is expressed by the familiarity of the experts with the selected indicators and the basis of their judgment. The quantitative values of the familiarity and judgment basis are shown in Tables 2, 3.

**Table 2:** Familiarity table for expert questionnaires.

**Degree of familiarity**	**Familiarity factor**
Very familiar	1
Familiar	0.8
Relatively familiar	0.6
Fair	0.4
Very unfamiliar	0.2

**Table 3:** Judgment basis of expert questionnaire indicators.

	**Impact degree factor**		
Basis of judgment	Large	Medium	Small
Theoretical level	0.8	0.6	0.4
Empirical level	0.6	0.4	0.2
Others	0.4	0.2	0.1
Intuitive level	0.2	0.1	0.1

A coefficient of authority value of ≥0.70 is generally considered to be within the acceptable range. In this study, Table 4 is derived from the statistical analysis of the authority of experts. The authority coefficients of the indicators are all above 0.80, which indicates that the members of the experts surveyed in this study have a high degree of authority.

**Table 4:** Statistical table of expert authority coefficient.

**Index**	**Judgment basis**	**Degree of familiarity**	**Degree of authority factor**
Training plan	0.86	0.80	0.83
Training implementation	0.90	0.88	0.89
Training monitoring	0.94	0.90	0.92
Work management	0.84	0.80	0.82
Competition performance	0.90	0.88	0.89

The degree of coordination of experts' opinions reflects the variability of the selected experts' evaluation of the index, which can be expressed by calculating the coefficient of variation and the coordination coefficient. The coefficient of variation is a statistical measure of the degree of variation of different experts' opinions, and the smaller the coefficient of variation, the higher the degree of coordination of experts. The formula is: CV = I/V, and CV represents the coefficient of variation, I represents the standard deviation, and V represents the mean. The expert coordination coefficient reflects the degree of consistency of experts' opinions on the index. In this study, the coordination coefficients and chi-square values of experts in the first and the second rounds were calculated based on the results of the first and the second rounds of expert consultation and the related formulae (Tables 5, 6).

**Table 5:** First round of expert coordination factor table.

**Index**	**Coordination factor**	**Chi-square**	***p***
Training plan	0.242	85.097	<0.01
Training implementation	0.365	150.952	<0.01
Training monitoring	0.327	157.860	<0.01
Work management	0.448	236.994	<0.01
Competition performance	0.556	327.557	<0.01
Total	0.263	483.793	<0.01

**Table 6:** Second round of expert coordination factor table.

**Index**	**Coordination factor**	**Chi-square**	***p***
Training plan	0.271	66.925	<0.01
Training implementation	0.447	190.127	<0.01
Training monitoring	0.486	203.164	<0.01
Work management	0.653	198.658	<0.01
Competition performance	0.675	180.331	<0.01
Total	0.391	534.621	<0.01

### Determine Indexes

In the form of written and online questionnaire survey, two rounds of survey were conducted among sports training experts, representative senior coaches, scientific research personnel, and training management personnel in the tennis industry. The final result was based on the second survey. According to the screening results, the importance degree (average score ≥4 points) is taken as the evaluation index of training quality of high-level tennis team.

### Reliability and Validity of the Questionnaire

The questionnaire was sent to 20 experts, and testing the validity of the questionnaire (Table 7) shows the (high) reasonability of the questionnaire content and structure design which meets the requirements of this study.

**Table 7:** Validity test table.

**Comprehensive evaluation**	**Very reasonable**	**Reasonable**	**General**	**Not very reasonable**	**Unreasonable**
Frequency	9	10	1	0	0
Percentage	45%	50%	5%	0	0

#### Questionnaire Validity Test

#### Questionnaire Reliability Test

In this study, we adopted the method of retest. After formally distributing the questionnaire for 15 days, 50% of the respondents were randomly selected to reissue the same questionnaire, and the data were processed. By comparing the results of the two surveys, the *r* = 0.835, *p* < 0.1 of the two questionnaires was calculated, indicating that the questionnaire issued in this study has high reliability.

### Determination and Analysis of Evaluation Index Weight

According to the requirements of analytic hierarchy process (AHP), this article designed a “questionnaire on the weight of elements in the hierarchical structure of training quality of high-level tennis teams.” SPSS25 was used for statistical analysis on the collected questionnaires, determining the weight of training quality indicators of high-level tennis teams, and analyzing the evaluation indicators.

## Experimental Results and Discussion

### Theoretical Interpretation of “Training Quality of High-Level Tennis Team”

#### Definition of “High-Level Tennis Team”

At present, there is no specific standard for judging the level of sports teams. In China, it is mainly used to implement athlete technical grading system (Table 8).

**Table 8:** Awarding standard of technical level for Chinese tennis players.

**Athlete level**	**Competition level**	**Event level**	**Award criteria**
World class athletes	Olympic Games, Australian Open, Wimbledon, French Open, us open, federation cup, Davis Cup, WTA, ATP	International events	According to the competition level and ranking
Master sportsman	Olympic Games, Australian Open, Wimbledon, French Open, American open, Youth Olympic Games, Davis Cup, WTA, ITF, World University Games	International events	
First-class athlete	Davis Cup, federation cup, world cup, ITF youth competition, Asian Youth Games, National Games, national championships, National Institutes of physical education, national college students competition	International and domestic events	

“*Technical grade standard for tennis players” revised by General Administration of Sport of China (2014)*.

At the present stage, the skill level of Chinese tennis players is mainly evaluated according to their competition level and ranking, which generally can be classified as elite athletes, first-class players, etc. But usually, due to the different recruitment standards and training conditions of sports teams in different provinces and cities, the overall competitive level is not judged by the individual performance of athletes, that is, the level of athletes themselves, but by the level of the competition and ranking of sports teams.

Some experts think that the “high level” is a relative concept. The training of high-level athletes should be divided into at least two levels: one level is the high level relative to the average student, where these athletes should have a high level of special sports skills, and this part of the sports team group with a certain foundation and skills is called “high level;” and another level is the high level relative to professional athletes, who have a good athletic talent and athletic foundation, professional athletes can be achieved through scientific training, and these athletes belong to the competitive type of “high level.” These two “high levels” play an indispensable role in the process of sports development, and they are also the embodiment of the training of sports talents at all levels (Yang and Song, 2005). Another groups of experts believe that high-level sports teams generally refer to provincial training teams and national training teams, which participate in competitions on behalf of the country, provinces, and cities, and finally achieve good rankings (Liu, 2007). In view of this, according to the above research, this article defined the level of high-level tennis team as based on the selection of competitive athletes, for the purpose of cultivating talents for competitive tennis, Asian Youth Games, national games, national championships, youth games, International Tennis Federation, and other provincial and municipal events that have certain qualifications.

#### Definition of “Training Quality”

The international organization for standardization defined quality as “the quality of things, products, or work” (International Organization for Standardization, 2015). Training quality is the lower concept, and there is a general and special relationship between them. Training quality involves sports, military science, special medicine, and other disciplines. In sports training, the broad sense of sports training quality refers to the development quality of the whole competitive sports, whereas the narrow sense of sports training quality mainly refers to the quality of special sports training process, that is, the comprehensive reflection of the quality of various links in the special sports training process (Hu, 2014, p. 309). The quality of sports training process known as the quality of training work, mainly includes the quality of special training decision-making, special training planning, special training design, special training implementation, and special training monitoring (Jiang, 2018). The evaluation of training work quality was the evaluation and measurement of the implementation, management, and monitoring of the internal links of the evaluation object's special sports training process through scientific evaluation methods according to the unified standards and certain evaluation procedures (Zhou and Tan, 2017).

Through the analysis of the training quality and training evaluation concept and the combination of reality characteristics of the High Level Tennis Team, we have consulted related sports training experts and senior coaches. We defined “the training quality” as: An activity that aims at achieve established training goals in a planned, organized, controlled, coordinated way through the adoption of scientific management and training methods. It specifically include: (1) training work planning, (2) training work implementation, (3) training work monitoring, (4) network team work management, and (5) sports competition results.

### Construction of Training Quality Evaluation System for High-Level Tennis Team

#### Evaluation Index Screening Process

**Figure 1:**
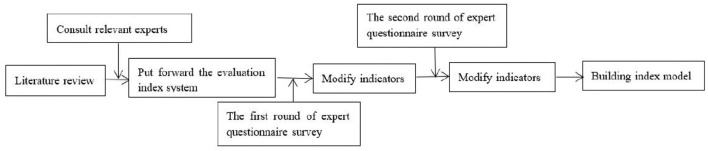
Index screening process.

To reflect and measure the research object comprehensively, the evaluation system is to reflect the characteristics and rules of high-level tennis team training, and the training quality evaluation index system can more comprehensively reflect the actual situation of high-level tennis team training. To ensure that the selected indexes can objectively and accurately reflect the training quality of high-level tennis teams, according to the relevant literature and existing research basis, preliminarily constructing the evaluation index system of tennis team training quality. After the screening, determining the evaluation index system of the training work of the high-level tennis team (Figure 1) is focused.

#### Primary Selection of Evaluation Index

Competitive ability includes five elements: physical fitness, skill, tactical ability, mental ability, and intelligence. Systematic training is a decisive factor for the steady improvement of athletes' competitive ability (Deng and He, 2018). Scientific training plan, reasonable training implementation, strict training monitoring, and perfect management system can promote athletes' mastery of tennis skills and tactics (Brzostowski and Szwach, 2018). Combined with the current working situation of high-level tennis team training, relevant literature, and experts' opinions, this article analyzed the planning, implementation, monitoring, management, and competition performance of high-level tennis teams, and about 197 indexes have been preliminarily formulated (Table 9).

**Table 9:** List of primary selection indexes for training quality evaluation of high-level tennis teams.

**Primary indicators**	**Secondary indicators**	**Third-level indicators**
Training plan of tennis team	Real state diagnosis	Diagnosis of players' physical fitness in non-competition period, Players in domestic competition cycle, Players' physical condition in ITF series, Players' physical function in non-competition period, Players' physical function in domestic competition cycle, Players' physical function in ITF series, Players' long-term psychological ability, Players' psychological ability before competition, In game and after the game, the diagnosis of players' psychological ability, The diagnosis of players' sports intelligence, The diagnosis of basic technical ability such as forehand and backhand stroke and serve, The diagnosis of tactical ability such as the change of serving net and return line, The diagnosis of doubles players' tacit understanding degree, The adaptability of field environment, The diagnosis of players' anti-interference ability, The diagnosis of physical distribution in the game, The diagnosis of domestic competition results, The diagnosis of competition performance of ITF series, Training load of non-competition period players, Training load diagnosis of domestic competition cycle players, Training load diagnosis of ITF series players, Analysis and diagnosis of competitors
	Training objective establishment	Players' physical function goals, Players' physical state goals, Forehand and backhand stroke and serve technical ability goals, Serve the net and return the ball line change tactical ability goals, Sports psychological goals, Sports intelligence goals, National competition performance goals, ITF series competition achievement goals, Multi-year training process testing objectives, Annual training process testing objectives, Phased training Process detection target
	Specific training content	Basic warm-up exercise, Serve training, Serve on the net mobile Footwork training, Serve and receive after the tactical convergence, Receive multiple ball training, Baseline to the front court forehand into the attack ball training, Forehand side body movement batting training, Baseline horizontal movement multi-ball training, two-person baseline counter stroke training, two-person forehand backhand baseline diagonal stroke training, “Before net baseline” round Stability training, Active volleying in front of the net, Large angle volleying in the middle and front court, 4 consecutive volleys in front of the net, Multi-ball training of high pressure ball in the middle field, Back stepping high pressure ball training in front of the field, Multi-ball training of volley in the middle field, Backhand cutting round training of 2 people, Hanging high ball return training in the middle and front field, 10 system singles match within the team, Cooperation training of doubles players Targeted situation simulation training, Team-specific physical training, ITF series of pre-competition psychological guidance, Sports intelligence training, Post-competition recovery training, Injury rehabilitation training
	Development of training plan	Multiyear training plan, Annual large cycle training plan, Non-competition period training plan, Domestic competition period phased training plan, ITF Series team training plan, Small cycle training course content formulation, Player Participation arrangement of all levels of singles and doubles events, Site type selection, Players' technical and tactical and special physical training design of different competition cycle, Training method selection of different competition cycle, Training load arrangement in different competition periods
Implementation of tennis team training	Guarantee of training conditions	The guarantee of ideological condition, System condition, Material condition, Medical condition, Logistic condition and Scientific research condition
	Training plan implementation	Team training course organization, Tennis technical and tactical training task completion degree, Athletes' special physical training activities, Specific guidance activities for single and double players, Special footwork training activities, Implementation of national competition training plan, Implementation of ITF series of events training plan, Athletes' recovery training activities, Athletes' rehabilitation training activities, Team training and scientific research combination
	Training process monitoring	During the non-competition period, The monitoring of players' Physical Fitness Standard, The adjustment of players' Physical function, The completion of basic techniques such as forehand and backhand hitting and serving, Serve the net and return the ball line change and other tactics, The monitoring of doubles players' cooperation degree, The monitoring of non-match players' psychological ability, The monitoring of players' psychological ability during the match period
	Training plan adjustment	Training task adjustment, training load adjustment, Training content adjustment, Training methods adjustment, Training environment adjustment, Training sequence adjustment, Training goal adjustment
Monitoring of tennis team training	Body function	Heart rate reserve, Blood lactic acid, Serum creatine kinase, Blood urea, Hemoglobin, Vital capacity
	Physical quality	Grip strength, 1-min push-up, Standing long jump, Vertical jump touch the ball, Sit-up, Squat with weight, Tennis throw in place, 1-min double swing rope skipping, Sitting start training, 30-m running, Side sliding of singles, 1-min forehand swing speed, Swivel pass, 400-m running, Half court back and forth run, Sitting body forward bending, Double line turn back run, Holding bat meter run, Left line forward sprint, Right line turn back sprint, Running, Forehand and backhand closed footwork throwing solid ball
	Sports tactics	First serve success rate, First serve scoring rate, Serve the net, Break success rate, Bottom line continuous change tactics, Repeated route change, Depth change, Over body ball tactics, Follow the ball net tactics
	Sports technology	Serve, Receive, Volley, High pressure, Baseline counter attack, Backhand chop, Reverse stroke, Cutting, Putting small ball, Contingency batting
	Psychological movement	Volitional quality, Emotional stability, Spatial perception, Hanoi Tower steps, Reaction time, Operation ability, self-confidence, Self-regulation and adaptability
Management of tennis team work	Training management of tennis team	Ideological education management, Training competition management, Athlete business management, Cultural learning management
	Organization and management of tennis team	Coach management, Athlete management, Training form management, Training task management, Training process management, Rules and regulations management, Medical team management, Nutrition and health management, Scientific research management
Sports competition results	Major competition results	World ranking, National ranking
	The trend of sports talents transportation	National team, National training team

#### The Optimization of Evaluation Index

Based on the primary indicators, the expert questionnaire was designed to evaluate the training quality indicators: very important (five points), important (four points), general important (three points), unimportant (two points), and very unimportant (one point). According to the results of expert questionnaire, the mean value and coefficient of variation of the indicators can be calculated. Among them, the mean value refers to the concentration degree of experts' opinions. The higher the value, the higher the importance, and the average value mean value is required to be not <4.0. The coefficient of variation is the coordination degree between experts on a certain index; the smaller the coefficient, the higher the coordination degree of the index (Zhou and Zhang, 2018), and the requirement is <0.2.

##### Survey Results and Analysis of First-Level Indexes

**Figure 2:**
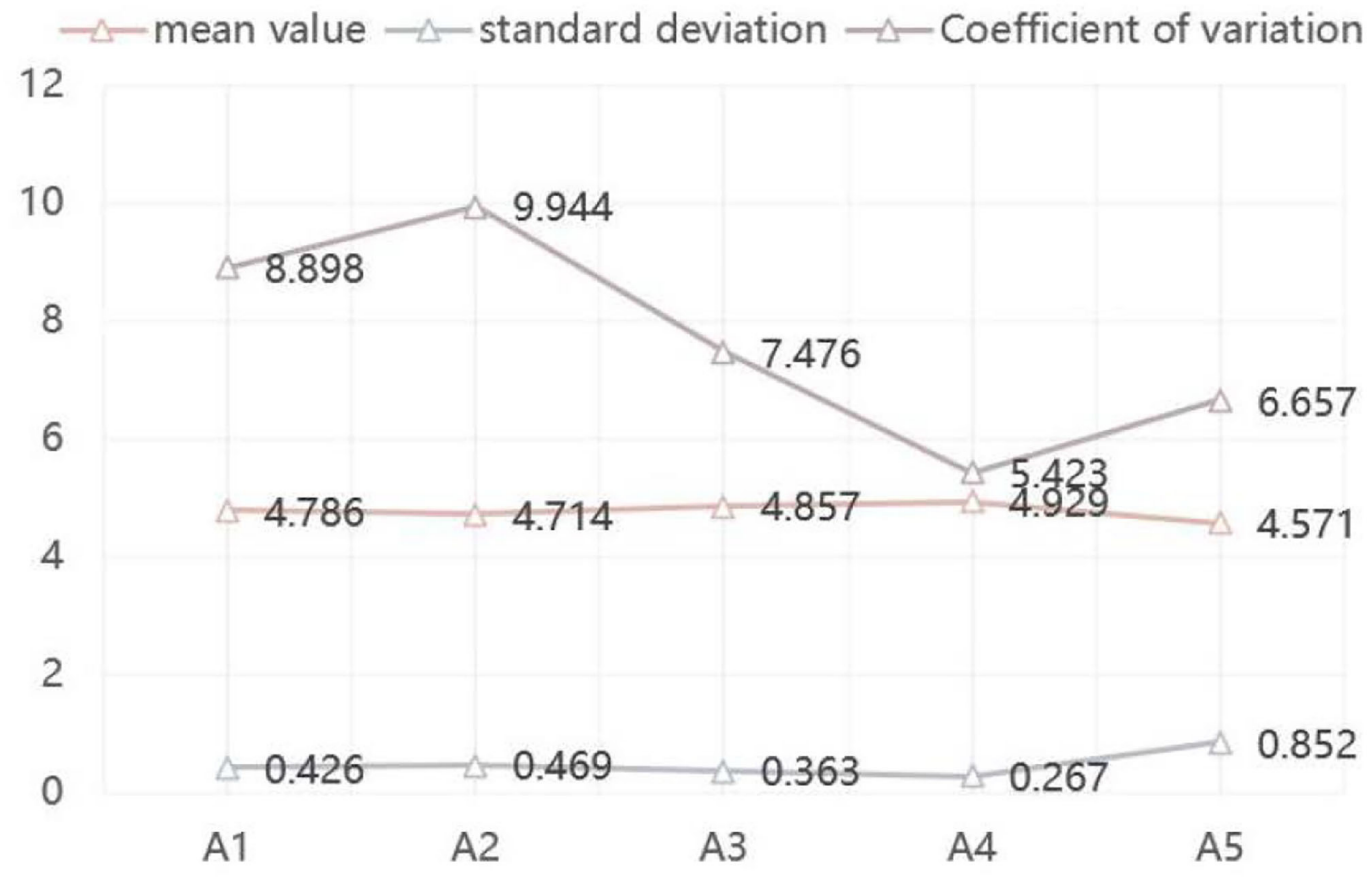
List of survey results of the first index experts.

Through the statistics and analysis of expert consultation results, deleting the indexes with mean value lower than 4.0 and higher coefficient of variation is implemented. It can be seen from Figure 2 and Table 10 that the importance scores of the first-level indexes are all >4.0, and meanwhile the coordination degree of expert consultation is also low (coefficient of variation is <0.20). It can be seen that the training quality of high-level tennis teams can be comprehensively evaluated from five aspects: training plan (A1), training implementation (A2), training monitoring (A3), work management (A4), and competition performance (A5).

**Table 10:** Survey results of first-level index experts.

**Secondary indicators**	**Mean**	**Standard**	**Coefficient of**
	**value**	**deviation**	**variation (%)**
A1 Training plan	4.786	0.426	8.898
A2 Training implementation	4.714	0.469	9.944
A3 Training monitoring	4.857	0.363	7.476
A4 Work management	4.929	0.267	5.423
A5 Competition performance	4.571	0.852	6.657

##### Investigation Results and Analysis of Second-Level Indexes

According to the results of expert consultation, this article analyzed the importance degree of the secondary indicators of the training quality of high-level tennis teams, and deleted the indicators whose importance degree score is lower than 4.0 and coordination degree coefficient of variation is higher than 0.2. It can be seen from Table 11 that the mean value of the secondary indexes are above 4.0, and the coefficient of variation is <0.2, indicating that experts agree with the 17 secondary indicators.

**Table 11:** Survey results of secondary index experts.

**Secondary indicators**	**Mean value**	**Standard deviation**	**Coefficient of variation (%)**
B1 Real state diagnosis	4.857	0.363	7.476
B2 Training objective establishment	4.857	0.363	7.476
B3 Specific content of training	4.929	0.267	5.423
B4 Training plan formulation	4.857	0.363	7.476
B5 Training Condition Guarantee	4.714	0.611	12.966
B6 Training plan implementation	4.929	0.267	5.423
B7 Training process monitoring	4.714	0.726	15.406
B8 Training plan adjustment	4.571	0.514	11.234
B9 Body function	4.357	0.842	19.322
B10 Physical fitness	4.929	0.267	5.423
B11 Sports tactics	5.000	0.000	0.000
B12 Sports technology	5.000	0.000	0.000
B13 Sports psychology	4.857	0.535	11.005
B14 Training management of	4.286	0.825	19.260
tennis team			
B15 Organization and management	4.286	0.825	19.260
of tennis team			
B16 Major competition results	4.929	0.267	5.423
B17 Trend of sports talents	4.714	0.726	15.406

##### Investigation Results and Analysis of the Third-Level Indexes

This article analyzed the importance degree of the three indexes of the training quality of high-level tennis teams and reserved the indexes whose importance degree score is higher than 4.0 and coordination degree coefficient of variation is lower than 0.2. Therefore, experts agree that the following 75 three-level indexes can accurately evaluate the quality of tennis team training and should be retained (Table 12). The deleted indicators are explained as follows: (1) experts think that in the process of index selection, some indexes information have overlapped; (2) evaluation indexes should focus on the implementation of training work, training work monitoring, and sports competition results; and (3) the quality evaluation of high-level tennis training should develop synchronously with tennis team training work, and some indexes evaluation has no differentiation.

**Table 12:** Indexes with “mean value” >4.0.

**Third-level index**	**Mean value**	**Standard deviation**	**Coefficient of variation**	**Third-level index**	**Mean value**	**Standard deviation**	**Coefficient of variation**
B11 Diagnosis of players' physical fitness in non-competition period and competition period	4.857	0.535	11.005	B82 Training load adjustment	4.286	0.825	19.260
B12 Diagnosis of players' physical function during non-competition period and during competition period	4.286	0.825	19.260	B83 Training content adjustment	4.857	0.363	7.476
B13 Diagnosis of players' psychological ability during competition	4.286	0.825	19.260	B84 Training method adjustment	4.533	0.516	0.1139
B14 Technical ability of forehand and backhand stroke and serve	4.857	0.363	7.476	B91 Heart rate reserve	4.733	0.458	0.0967
B15 Serve the net and return the ball line change and other tactics	4.533	0.516	0.1139	B92 Hemoglobin	4.933	0.258	0.0523
B16 Diagnosis of training load of players in non-competition period and competition period	4.6	0.507	0.1102	B93 Blood lactic acid	4.4	0.632	0.1437
B21 Physical fitness goals of players	4.667	0.488	0.1046	B94 Grip strength	4.8	0.414	0.0863
B22 Technical ability of forehand and backhand stroke and serve	4.733	0.458	0.0967	B101 1 minute push up	4.867	0.352	0.0723
B23 Serve the net and return the ball line change and other tactics	4.933	0.258	0.0523	B102 Standing long jump	4.267	0.704	0.1649
B24 National competition and ITF series competition results	4.4	0.632	0.1437	B103 Head toss	4.467	0.516	0.1156
B25 Target detection in training process	4.667	0.488	0.1046	B104 100M run	4.667	0.488	0.1046
B31 Basic warm-up exercises	4.733	0.458	0.0967	B105 Changing line sliding in singles	4.733	0.458	0.0967
B32 Technical and tactical training of serving and receiving	4.8	0.414	0.0863	B106 1 min swing speed	4.4	0.507	0.1153
B33 Forward and backhand attack ball training from baseline to front court	4.733	0.458	0.0967	B107 400M run	4.867	0.352	0.0723
B34 Volley training in front of the net	4.8	0.414	0.0863	B108 Sit forward	4.933	0.258	0.0523
B35 High pressure ball multi-ball training in midfield	4.867	0.352	0.0723	B109 100M run	4.8	0.414	0.0863
B36 Psychological guidance of ITF series and other events	4.267	0.704	0.1649	B111 Serve tactics	4.933	0.258	0.0523
B37 Post-game recovery and injury rehabilitation training	4.467	0.64	0.1432	B112 Receiving tactics	4.933	0.258	0.0523
B41 Development of training tasks for non-competition period and competition period	4.533	0.516	0.1139	B113 Baseline tactics	4.533	0.516	0.1139
B42 Training contents of domestic competition period and ITF series	4.467	0.516	0.1156	B114 Tactics in front of the net	4.667	0.488	0.1046
B43 Technical tactics and physical fitness of players in different competition periods	4.667	0.488	0.1046	B121 Service techniques	4.733	0.458	0.0967
B44 Training load arrangement in different competition periods	4.733	0.458	0.0967	B122 Service receiving technique	4.8	0.414	0.0863
B51 Guarantee of ideological conditions	4.4	0.507	0.1153	B123 Volley technique	4.733	0.458	0.0967
B52 Material condition guarantee	4.8	0.414	0.0863	B124 High pressure technology	4.8	0.414	0.0863
B53 Medical condition guarantee	4.933	0.258	0.0523	B125 Baseline counter attack technology	4.667	0.488	0.1046
B54 Guarantee of scientific research conditions	4.933	0.258	0.0523	B131 Will quality	4.267	0.799	0.1872
B61Team training course organization	4.533	0.516	0.1139	B132 Emotional stability	4.933	0.258	0.0523
B62 Tennis skills and tactics training completion	4.6	0.507	0.1102	B133 Self-confidence	4.933	0.258	0.0523
B63 Targeted guidance activities for single and double players	4.933	0.258	0.0523	B141 Ideological education management	4.6	0.507	0.1102
B64 Training plan for national events and ITF series events	4.867	0.352	0.0723	B142 Training competition management	4.6	0.507	0.1102
B63 Targeted guidance activities for single and double players	4.933	0.258	0.0523	B141 Ideological education management	4.6	0.507	0.1102
B64 Training plan for national events and ITF series events	4.867	0.352	0.0723	B142 Training competition management	4.6	0.507	0.1102
B65 Training activities of athletes in recovery period and rehabilitation period	4.933	0.258	0.0523	B143 Athlete business and culture	4.6	0.507	0.1102
B66 Combination of team training and scientific research	4.8	0.414	0.0863	B151 Coach management	4.8	0.414	0.0863
B71 Monitoring of players' physical fitness in non-competition period	4.733	0.458	0.0967	B152 Athlete management	4.867	0.352	0.0723
B72 Physical adjustment of players	4.4	0.507	0.1153	B153 Training process management	4.733	0.458	0.0967
B73 Complete the basic skills of forehand and backhand hitting and serving	4.4	0.632	0.1437	B161World rankings	4.933	0.258	0.0523
B74 Serve the net and return the ball line change and other tactics	4.667	0.488	0.1046	B162 National ranking	4.867	0.352	0.0723
B75 Psychological monitoring of players in non-competition period and competition period	4.733	0.458	0.0967	B171 National team	4.933	0.258	0.0523
B81 Training task adjustment	4.8	0.414	0.0863	B172 National training team	4.867	0.352	0.0723

#### Consistency Test of Statistical Results of Expert Survey

In the selection process of the evaluation indexes of the training quality of high-level tennis team, experts have deviation on the tennis team training work, and the index evaluation results may be inconsistent. Therefore, the validity of the evaluation results of the consistency test of experts' opinions is expressed by the coefficient of variation (V) and the coefficient of coordination (W). The smaller the variation coefficient, the higher the consistency; when the value of the coordination coefficient is between 0 and 1, the greater the value, the higher the degree of consistency.

Coefficient of variation formula: $V=\frac{\partial }{\bar{X}}$ (∂ is the standard deviation; $\bar{X}$ is the mean value).

Coordination coefficient formula: $W=\frac{12\overset{k}{\underset{i=1}{\sum }}Ri-3b^{2}k\left(k+1\right)}{b^{2}k\left(k^{2}-1\right)}$ (*b* is the number of experts; *k* is the number of evaluation indexes).

The results of expert questionnaire survey: using SPSS25 to analyze the expert evaluation results, the results show that the consistency coefficient of the first-level index is 0.688, the second-level index is 0.479, and the third-level index is 0.744; *p* < 0.01. Therefore, it is considered that the experts have a good consistency in the evaluation indexes of the training quality of high-level tennis teams, and the evaluation result is credible.

### Determination of Training Quality Evaluation Indexes for High-Level Tennis Teams

According to experts' opinion concentration and coordination degree to eliminate, merge, modify, and delete indicators, the average index score above four points, expert opinion concentration and overall coordination degrees below 0.2 shall be preserved. Finally, the evaluation indexes of training quality of high-level tennis teams, including five first-class indexes, 17 second-class indexes, and 75 third-class indexes is determined (Table 13).

**Table 13:** Quality evaluation index list of high-level tennis team trainers.

**Primary indicators**	**Secondary indicators**	**Third-level indicators**
A1 Training plan of tennis team	B1 Reality diagnosis	B11 Diagnosis of players' physical fitness in non-competition period and competition period, B12 Diagnosis of players' physical function in non-competition period and competition period, B13 Diagnosis of players' psychological ability during competition, B4 Diagnosis of basic technical ability of forehand and backhand stroke and serve, B15 Diagnosis of the tactical ability of serving, getting on the net and changing the return line, B16 Diagnosis of training load of players in non-competition period and competition period
	B2 Training objective establishment	B21 Physical fitness goals of players, B22 National competition and ITF series competition achievement goal, B23 The goal of tactical ability, such as the change of serve, net and return line, B24 Technical ability target of forehand and backhand stroke and serve, B25 Target detection in training process
	B3 Specific training content	B31 Technical and tactical training of serving and receiving, B32 Forward and backhand attack ball training from baseline to front court, B33 Volley training in front of the net, B34 High pressure ball multi ball training in midfield, B35 Psychological guidance of ITF series and other events, B36 Post-game recovery and injury rehabilitation training
	B4 Training plan formulation	B41 Development of training tasks for non-competition period and competition period, B42 Training content formulation of domestic competition period and ITF series, B43 Technical tactics and special physical training design of players in different competition periods, B44 Training load arrangement in different competition periods
A2 Implementation of tennis team training	B5 Guarantee of training conditions	B51 Ideological condition guarantee, B52 Material condition guarantee, B53 Medical condition guarantee, B54 Scientific research condition guarantee
	B6 Training plan implementation	B61 Team training course organization, B62 Tennis skills and tactics training completion, B63 Targeted guidance activities for single and double players, B64 Implementation of training plan for national events and ITF series events, B65 Training activities of athletes in recovery period and rehabilitation period, B66 Combination of team training and scientific research
	B7 Training process monitoring	B71 Monitoring of players' physical fitness in non-competition period, B72 Physical adjustment of players, B73 Completion of basic techniques of forehand and backhand hitting and serving, B74 serves t tactical utilization situation of the net and returns the ball the line change and so on, B75 Psychological monitoring of players in non-competition period and competition period
	B8 Training plan adjustment	B81 Training task adjustment, B82 Training load adjustment, B83 Training content adjustment, B84 Training method adjustment
A3 Monitoring of tennis team training	B9 Special body function	B91 Heart rate reserve, B92 hemoglobin, B93 Blood lactic acid
	B10 Special physical fitness	B101 Grip strength, b102 1 min push-up, B103 Standing long jump, B104 Head up throw solid ball, B105 100M run, B106 Singles variable line sliding step, b107 1 min swing speed, b108 400M run, B109 sit forward bend
	B11 Special sports tactics	B111 Serving tactics, B112 Receiving tactics, B113 Baseline tactics and B114 Pre net tactics
	B12 Special sports technique	B121 Service technology, B122 Receiving technology, B123 Volley technology, B124 high pressure technology, B125 Baseline counter attack technology
	B13 Special sports psychology	B131 Will quality, B132 Emotional stability, B133 Self-confidence
A4 Management of tennis team work	B14 Training management of tennis team	B141 Ideological education management, B142 Training competition management, B143 Athlete business and cultural learning management
	B15 Organization and management of tennis team	B151 Coach management, B152 Athlete management, B153 Training process management
A5 Sports competition results	B16 Major competition results	B161 World ranking, B162 National ranking
	B17 The trend of sports talents transportation	B171 National team, B172 National training team

#### Determination of Weight Coefficient of Evaluation Indexes

In this study, the AHP was mainly used to determine the weight coefficients of the evaluation indexes, which is not only a multi-objective evaluation decision-making method suitable for complex structure but also an evaluation method combining qualitative and quantitative analyses. It is decomposed into general objective layer, sub-objective layer, and criterion layer according to the leading relationship of factors, divided into general target layer, sub-target layer, and criterion layer, constructing the multi-target and multi-level model to form a progressive hierarchical structure. By calculating the importance of the low-level evaluation matrix, one yields the importance ranking of the low-level evaluation matrix (Satti, 1988).

#### Establish Hierarchical Structure Model

**Figure 3:**
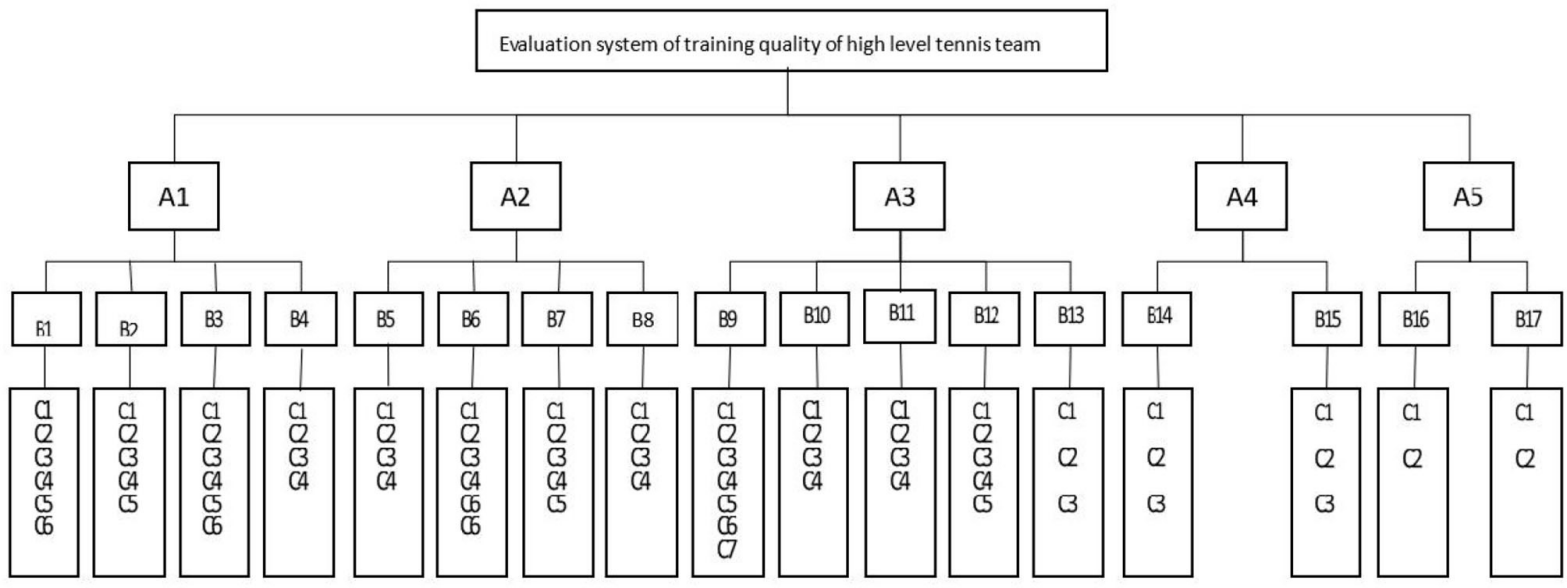
Hierarchy chart of training quality evaluation for high-level tennis team.

From the perspective of high-level tennis team training quality evaluation, through consulting a large number of studies at home and abroad, it can be found that it mainly includes five parts: training work planning, training work implementation, training work monitoring, sports competition results, and sports team work management (Figure 3 shows the hierarchical structure chart of quality evaluation of training work of high-level tennis teams).

#### Constructing Pairwise Judgment Matrix

In constructing a progressive hierarchy, each element and the next layer of elements dominated by the same layer constitute a sub-region, and an expert survey is conducted on the indicators of this sub-region to construct a judgment matrix diagram.


(1) $$\begin{array}{l} A={\left(b_{ij}\right)}_{n\times n}=\left\|\begin{array}{l} B_{11}B_{12}B_{13}.........B_{1n}\\ B_{21}B_{22}B_{23}........B_{2n}\\ ..................\\ B_{n1}B_{n2}B_{n3}.........B_{nn} \end{array}\right\| \end{array}$$

*Bij* represents the relative importance of each element. Generally, the logic judgment of relative importance is quantified by 1–9 scale method, and the value is given according to the comprehensive judgment of experts (Table 14).

**Table 14:** Assignment rules of analytic hierarchy process.

**Sequence number**	**Relative importance**	**Bij**	**Sequence number**	**Relative importance**	**Bij**	**Sequence number**	**Relative importance**	**Bij**
1	Bi is as important as Bj	1	4	Bi and Bj are strongly important	7	7	Bi and Bj are obviously not important	1/5
2	Bi is slightly more important than Bj	3	5	Bi and Bj are extremely important	9	8	Bi and Bj are strongly unimportant	1/7
3	Bi is more important than Bj	5	6	Bi and Bj are not important	1/3	9	Bi and Bj are extremely unimportant	1/9

##### Sorting Single Level

The hierarchical single ranking is a matrix diagram based on the working quality of the high-level tennis team training. It calculates the eigenvalues and eigenvectors of the matrix, and its specific calculation steps are as follows:

- Calculate the product of each row in the judgment matrix:

(2) $$\begin{array}{l} M_{i}=\overset{n}{\underset{i=1}{\prod }}C_{ij}.\left(i=1.2.3.....n\right) \end{array}$$

- Calculate the *n*th root w of each row M:

(3) $$\begin{array}{l} W_{X}=\sqrt[{\text{n}}]{Mi} \end{array}$$

- Calculate the different index weights:

(4) $$\begin{array}{l} {\overset{\wedge }{W}}_{i}=\frac{\bar{w_{i}}}{\overset{n}{\underset{j=1}{\sum }}\bar{w_{i}}}\left(j=1,2,3....n\right) \end{array}$$

Where W_i_ is the approximate value of eigenvector, that is, the weight of each factor.

- Calculate the maximum eigenvalue of the judgment matrix graph λ_max_:

(5) $$\begin{array}{l} \lambda_{\max}=\frac{1}{n}\overset{n}{\underset{i=1}{\sum }}\frac{\left(A\overset{\wedge }{w}\right)i}{wi} \end{array}$$

Where ${\left(A\overset{\wedge }{W}\right)}_{i}$is the i element of vector $A\overset{\wedge }{W}$.

When the relative weight of the first-level index is obtained, the next-level index weight is calculated. There is a hierarchical model composed of target Z, the first-level index layer a, the second-level index layer B, and the third-level index layer C. The relative weight of target layer Z to the first-level index a is as follows:


(6) $$\begin{array}{l} \bar{w}={\left(w_{1},w_{2},....w_{k}\right)}^{T},i=1,2,3,....,k \end{array}$$

The weight of index B in the second-level index layer is as follows:


(7) $$\begin{array}{l} \bar{w_{i}}={\left(w_{1i},w_{2i},....,w_{ni}\right)}^{T},i=1,2,3...,k \end{array}$$

The weight of C index in the third-level index layer is as follows:


(8) $$\begin{array}{l} {\bar{w}}_{ik}={\left(w_{1ik},w_{2ik},......,w_{nik}\right)}^{T},i=1,2,3.....k \end{array}$$

According to the above steps, the weight of training quality index system of high-level tennis team can be determined finally.

##### Consistency Test

- Calculate consistency index CI: *CI* = (λ max − *n*)/(*n* − 1)
- Consistency ratio CR: *CR* = *CI*/*RI* (RI values are shown in Table 15)

It can be seen that the random consistency index CR < 0.1; the smaller the Cr, the better the consistency of the judgment matrix. Generally speaking, when CR < 0.1, the judgment matrix meets the consistency test; otherwise, the judgment matrix should be adjusted appropriately. Through the above steps, the weight of each index of training quality of high-level tennis team is obtained. At the same time, through the consistency test (CR < 0.1), the index weight of training quality of high-level tennis team is as shown in Table 16.

**Table 15:** RI value of partial random consistency index.

**Number of elements**	**1**	**2**	**3**	**4**	**5**	**6**	**7**	**8**	**9**
9	0.00	0.00	0.58	0.90	1.12	1.24	1.32	1.41	1.46

**Table 16:** List of evaluation index system and weight of training quality of high-level tennis team.

**First-level index and weight**	**Secondary index and weight**	**Third-level index and weight**
	Real state diagnosis 0.10 (0.018)	Diagnosis of players' physical fitness in non-competition period and competition period 0.06 (0.0011), Diagnosis of training load of players in non-competition period and competition period 0.11 (0.0020), Diagnosis of players' psychological ability during competition 0.12 (0.0021), Diagnosis of basic technical ability of forehand and backhand stroke and serve 0.30 (0.0054), Diagnosis of the tactical ability of serving, getting on the net and changing the return line 0.26 (0.0046), Diagnosis of players' physical function in non-competition period and competition period 0.15 (0.0027)
	Training goal establishment 0.30 (0.054)	Physical fitness goals of players 0.06 (0.0032), National competition and ITF series competition achievement goal 0.42 (0.0227), The goal of tactical ability, such as the change of serve, net and return line 0.26 (0.0140), Technical ability target of forehand and backhand stroke and serve 0.16 (0.0086), Target detection in training process 0.10 (0.0054)
Tennis team training plan 0.18	Training content 0.38 (0.0684)	Technical and tactical training of serving and receiving 0.25 (0.0171), Forward and backhand attack ball training from baseline to front court 0.23 (0.0157), Volley training in front of the net 0.21 (0.0144), High pressure ball multi-ball training in midfield 0.14 (0.0096), ITF series and other events psychological guidance 0.09 (0.0062), Post-game recovery and injury rehabilitation training 0.08 (0.0055)
	Training plan making 0.22 (0.0396)	Development of training tasks for non-competition period and competition period 0.30 (0.0119), Training content formulation of domestic competition period and ITF series 0.20 (0.0079), Technical tactics and special physical training design of players in different competition periods 0.28 (0.0111), Training load arrangement in different competition periods 0.22 (0.0087)
	Training condition guarantee 0.20 (0.040)	Ideological Condition Guarantee 0.48 (0.0192), material condition guarantee 0.12 (0.0048), medical condition guarantee 0.22 (0.0088), scientific research condition guarantee 0.18 (0.0072)
	Training plan implementation 0.37 (0.074)	Team training course organization 0.20 (0.0148), Tennis skills and tactics training completion 0.29 (0.0215), Targeted guidance activities for single and double players 0.16 (0.0118), Implementation of training plan for national events and ITF series events 0.13 (0.0096), Training activities of athletes in recovery period and rehabilitation period 0.14 (0.0104), Combination of team training and scientific research 0.08 (0.0059)
Implementation of tennis team training 0.20	Training process monitoring 0.15 (0.03)	Monitoring of players' physical fitness in non-competition period 0.20 (0.006), Physical adjustment of players 0.25 (0.0075), Completion of basic techniques of forehand and backhand hitting and serving 0.20 (0.006), The tactical application of serve, net and return line 0.25 (0.0075), Psychological monitoring of players in non-competition period and competition period 0.10 (0.003)
	Training plan adjustment 0.28 (0.056)	Training task adjustment 0.20 (0.0112), Training load adjustment 0.37 (0.0207), Training content adjustment 0.15 (0.0084), Training method adjustment 0.28 (0.0157)
	Physical function 0.20 (0.06)	Heart rate reserve 0.30 (0.018), hemoglobin 0.54 (0.0324), blood lactate 0.16 (0.0096)
	Physical fitness 0.25 (0.075)	Grip strength 0.09 (0.0068), 1 min push up 0.08 (0.006), Standing long jump 0.12 (0.009), Spot tennis throw 0.11 (0.0082), 100-m run 0.12 (0.009), Singles side line sliding step 0.13 (0.0098), 1-min swing speed 0.10 (0.0075), 400-m run 0.13 (0.0098), Sit forward 0.12 (0.009)
Tennis team training work monitoring 0.30	Sport tactics 0.20 (0.06)	Serve tactics 0.30 (0.018), Receiving tactics 0.20 (0.012), Baseline tactics 0.28 (0.0168), Tactics in front of the net 0.22 (0.0132)
	Sports technique 0.25 (0.075)	Service techniques 0.20 (0.015), Service receiving technique 0.29 (0.0218), Volley technique 0.16 (0.0122), High pressure ball technology 0.13 (0.0023), Lob technique0.14 (0.0107), Baseline counter attack technology 0.08 (0.006)
	Sports psychology 0.10 (0.03)	Will quality 0.40 (0.012), Emotional stability 0.40 (0.012), Self-confidence 0.20 (0.006)
Sports team work management 0.12	Tennis team training management 0.50 (0.06)	Ideological education management 0.40 (0.024), Training and competition management 0.40 (0.024), Athletes' business and cultural learning management 0.20 (0.012)
	Organization and management of tennis team 0.50 (0.06)	Coach management 0.30 (0.003), Athlete management 0.54 (0.0324), Training process management 0.16 (0.0096)
Sports competition results 0.20	Major competition score 0.67 (0.134)	World ranking 0.67 (0.0898), National ranking 0.33 (0.0442)
	Trend of sports talents 0.33 (0.066)	National team 0.63 (0.0.04157), National training team 0.37 (0.0024)

*The brackets in the column are the weights of the second- and the third-level indexes, and the data are the results after normalization, that is, the results of the weights of the first-level indexes and the second-level indexes, and the results of the weights of the second- and the third-level indexes*.

**Figure 4:**
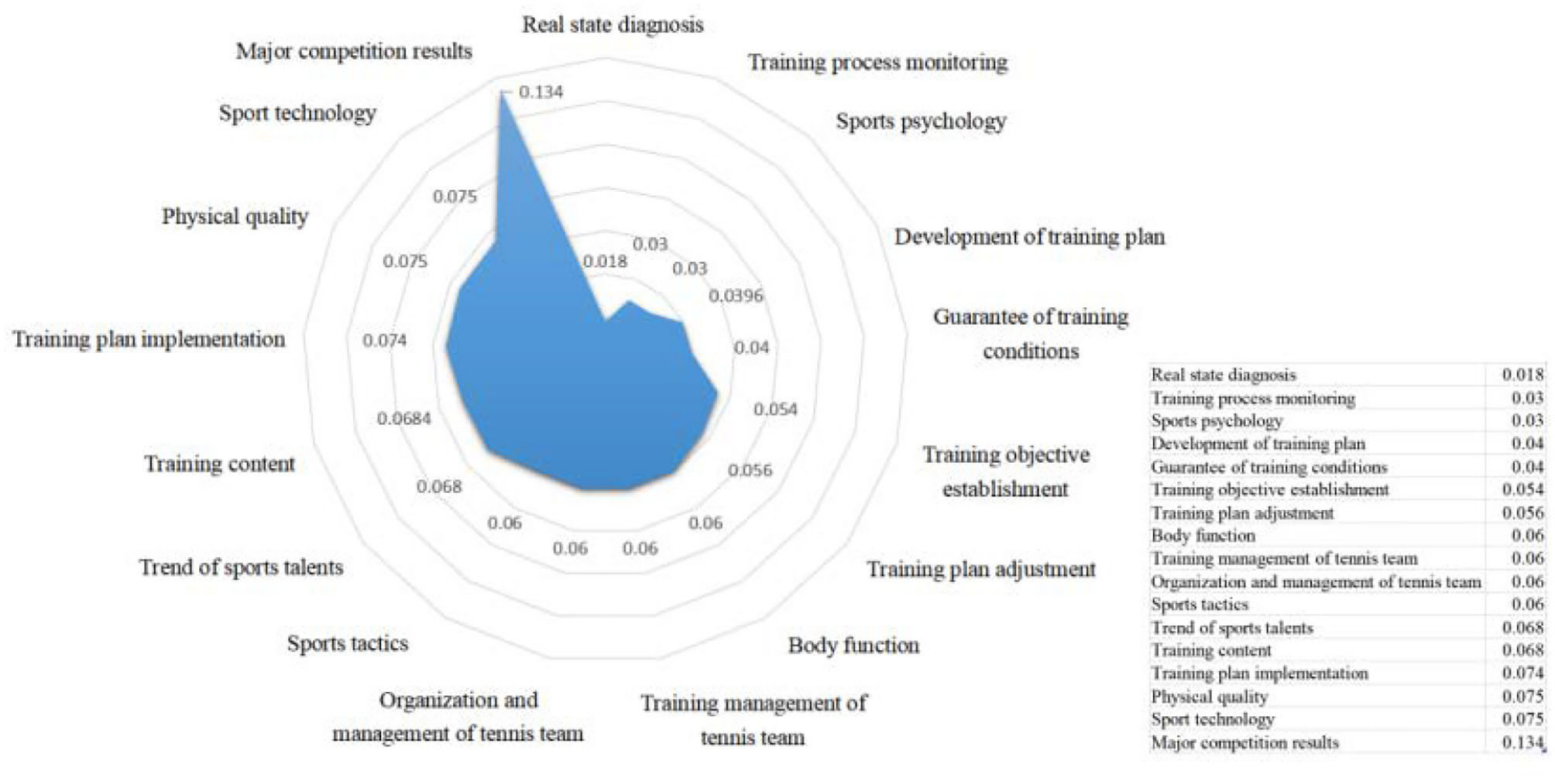
Ranking order of secondary index weight in training quality evaluation of high-level tennis team.

The secondary index weight of training quality of high-level tennis team is sorted from large to small according to the normalized processing results (Figure 4) comprehensive planning is the basis and guidance of tennis teams training for high-level tennis teams to carry out training work. The core is to form comprehensive training and master tennis skills and tactics by combining training implementation with monitoring (Penalva et al., 2022). It is key to strengthen the management of tennis team work, which is consistent with the scientific idea of improving the quality of training work.

### Index Analysis of Training Quality of High-Level Tennis Team

#### Analysis on Evaluation Index of Tennis Team Training Plan

Training planning is a process in which a high-level tennis team formulates training contents and plans according to the players' actual state and phased training objectives. Scientific and systematic training plan is not only the blueprint of tennis team training, but also the main carrier of the whole training work implementation (National Strength Conditioning Association, 2012). In the evaluation index system of tennis team training quality, the training work planning includes four secondary indexes: real state diagnosis (0.10), training goal establishment (0.30), training specific content (0.38), and training plan formulation (0.22). As the starting link of tennis team training, the real-time state diagnosis provides an important basis for the integration analysis and judgment of players' real-time status, the establishment of training objectives, and the implementation of training plans and contents. Combined with the actual situation of players, the establishment of training objectives is a process of systematic and reasonable design of phased work objectives according to the cycle characteristics of tennis training and competition. The specific content of training mainly refers to the sum of the elements that constitute the competitive ability of players in the process of sports training (Francisco et al., 2022). At the same time, the sports team will make appropriate adjustments to the players according to the competition cycle, whereas the formulation of training plan refers to the theoretical design of training activities before the start of sports training process. By dividing training content into several specific tasks closely related to each other, to ensure the whole sports personnel participate in the training (Dou and Sun, 2019), so as to effectively control the process of sports training.

#### Analysis of the Implementation of the Tennis Team Training

The implementation of training work is the concrete display of the whole training work. which should not only consider the intricate internal factors, but also consider the operability of the actual situation (Nicola and Keith, 2022). In the tennis team training quality evaluation index system, the training implementation mainly includes four indicators: training condition guarantee (0.20), training plan implementation (0.37), training process monitoring (0.15), and training plan adjustment (0.28). The guarantee of training conditions is the basis for the smooth development of sports team training. Only by checking and improving the physical and medical conditions of the tennis team, we can ensure the normal training of the tennis team (Li et al., 2021). Based on the guarantee of the training conditions, the training plan is implemented on the actual situation. Combined with the training characteristics of the tennis team and the requirements of the training plan, the systematic training for player can be implemented. The training process monitoring mainly refers to the coaches' recording and comparative analysis according to the actual situation of the training work, and feedback of the training effect to the players, which played a guiding role in adjusting the player's training plan and improving the training quality (Alan, 1981). The training plan adjustment is essentially the coach's pertinence to the original plan according to the players' training status and competition tasks so as to achieve the phased training goals of players. It is an indispensable part of tennis team training (Sudo and Souza, 2012).

#### Analysis on Monitoring and Evaluation Index of Tennis Team Training

Training work monitoring is not only the real-time supervision and feedback of training effect, but also the action guide for dynamic adjustment of coaches' strategies. Whether the training work planning is appropriate, the training work is scientific, the sports team management is comprehensive, and the sports competition results meet the expectations, etc., need to be fed back according to the training work monitoring, so as to provide scientific and effective reference for the player training work (Yu, 2015). From this it can be seen that the monitoring of training is the key to the training of tennis team, which is closely connected with all aspects of training. In the tennis team training quality evaluation index system, the training work monitoring occupies an important position, mainly including physical function (0.20), physical fitness (0.25), sports tactics (0.20), sports technology (0.25), and sports psychology (0.10). Physical function monitoring is the key factor to ensure the tennis players to achieve the best load state in the training process. Good physical quality is the basis for the follow-up development of high-level tennis players, mastering difficult movements, undertaking heavy load training, and fierce competition (Roetert, 2011). Skills and tactics are the skills that tennis players complete the competition with the most representative, optimal, and scientific overall action mode or behavior strategy under the corresponding sports ability. It is the core of training work monitoring. Therefore, the sports psychology is combined with the tennis skill and tactics training and the physical quality training, which runs through the whole training and competition of players (Adrian, 2003).

#### Analysis on the Evaluation Index of Tennis Team Work Management

The work management of tennis team is the premise and foundation to improve and guarantee the training work, and is a systematic project to establish and maintain the implementation of the work (Nancy, 2001). In the high-level tennis team training quality evaluation system, including sports training management (0.50) and sports organization management (0.50) two second-level indexes. Tennis team training management is an effective integration of sports training resources, so as to standardize the training process and improve the quality of tennis team training. Scientific sports training management procedure is the core of excellent sports team management, which is in the central position in the management of tennis team. The organization and management of tennis team can promote the mutual cooperation among coaches, athletes, tennis team and relevant departments, and the organization and management personnel. It can also promote personal management to the level of comprehensive management (Zhang, 2018). In the management of tennis team work, it is necessary to improve the scientificity of tennis training management and organization management. Through the mutual integration of the two, the management of tennis team can be completed together, and then the overall strategic goal can be achieved (Carlos, 2003).

#### Analysis on the Evaluation Index of Sports Competition Performance

Sports competition performance is a characteristic symbol reflecting the result of training work, but also an important symbol of evaluating the quality of training work (Vescovi, 2017). The training quality of high-level tennis team is presented to the public in the form of sports competition. The result of sports competition is the final basis for assessing the overall effectiveness of training work, and plays a leading role in the overall training work (Machar et al., 2016). In the evaluation index system of tennis team training quality, the sports competition results mainly include two second-level indexes: the major competition result (0.67) and the trend of sports talents (0.33). Major competition results are the ultimate goal of tennis players to participate in the competition, and it is also a comprehensive evaluation of the competitive ability of players and opponents in the competition (Machar et al., 2007). The evaluation not only includes the result and ranking of tennis players, but also includes the competitive skill level displayed by the players in the competition. The trend of sports talents must be combined with the competitive level and performance of tennis players according to the actual situation. Therefore, in order to ensure the scientific evaluation of sports competition results, the competitive level and the position of athletes should be evaluated from the competition results, and the trend of athletes should be evaluated from the action, so as to continuously improve the competitive ability of athletes (Clarke, 2011).

## Conclusion

### Concept Definition

The concept of “training quality of high-level tennis team” is preliminarily defined, which mainly includes five aspects: training work planning, training work implementation, training work monitoring, sports competition results, and sports team work management.

### Indicator Screening and Construction

Constructing the evaluation system of training quality of high-level tennis team included 5 first-class indexes, 17 second-class indexes, and 75 third-class indexes. In addition, formulating the weight table of training quality evaluation indexes for high-level tennis teams.

### Formulate the High-Level Net Team Training Quality Components

This study analyzed the main components of the training quality of high-level tennis team as follows: training work planning is not only the blueprint of tennis team training, but also the main carrier of the implementation of the whole training work. The implementation of training work is the concrete display of the whole training work, which should not only consider the intricate internal factors, but also consider the operability of the actual situation. Training work monitoring is not only the real-time supervision and feedback of training effect, but also the action guide for dynamically adjusting coaches' policies. Sports competition performance is a characteristic symbol of reflecting the result of training work and an important symbol to evaluate the quality of training (Gema et al., 2011). The management of tennis team is the premise and foundation to improve and guarantee the training work, and it is a systematic project to establish and maintain all the work.

### The Purpose of the Index System Construction

The evaluation index system of training quality of high-level tennis team constructed in this research can help the coaches to improve and adjust the training control factors and to promote the comprehensive and coordinated development of high-level athletes. At the same time, the evaluation criteria for the follow-up training work promote the sustainable development of China's competitive tennis industry and ensure the realization of the overall development goals of China's competitive sports.

## Expectation

In the follow-up work, based on the index system and weight of this study, the comprehensive evaluation standards will be formulated and then applied in some high-level tennis teams. In order to provide theoretical and practical guidance for the development of high-level tennis team guidelines, forming a set of high-level tennis team training quality evaluation system by screening and repairing the problems in the actual operation is essential.

In the future research, comprehensive analysis and evaluation model of training quality will be built through which we can realize the quantitative analysis, diagnosis, and evaluation of various types and constituent factors of training quality of high-level tennis teams. According to the analysis and evaluation results, the work quality composition chart of high-level tennis team will be established, so as to carry out scientific monitoring on the sports training process to achieve the purpose of “promoting quality by evaluation.”

## Data Availability Statement

The original contributions presented in the study are included in the article/supplementary material, further inquiries can be directed to the corresponding author.

## Author Contributions

All authors listed have made a substantial, direct, and intellectual contribution to the work and approved it for publication.

## Funding

This study was supported by Scientific Research Project of Hubei Provincial Department of Education, project name: Study on Quality Monitoring and Evaluation of Training of High-level Professional Young Female Tennis Team in China.

## Conflict of Interest

The authors declare that the research was conducted in the absence of any commercial or financial relationships that could be construed as a potential conflict of interest.

## Publisher's Note

All claims expressed in this article are solely those of the authors and do not necessarily represent those of their affiliated organizations, or those of the publisher, the editors and the reviewers. Any product that may be evaluated in this article, or claim that may be made by its manufacturer, is not guaranteed or endorsed by the publisher.
